# Activation of 2,4-Diaminoquinazoline in *Mycobacterium tuberculosis* by Rv3161c, a Putative Dioxygenase

**DOI:** 10.1128/AAC.01505-18

**Published:** 2018-12-21

**Authors:** Eduard Melief, Shilah A. Bonnett, Edison S. Zuniga, Tanya Parish

**Affiliations:** a TB Discovery Research, Infectious Disease Research Institute, Seattle, Washington, USA

**Keywords:** *Mycobacterium tuberculosis*, antitubercular, diaminoquinazoline, dioxygenases, monooxygenases, prodrug

## Abstract

The diaminoquinazoline series has good potency against Mycobacterium tuberculosis. Resistant isolates have mutations in Rv3161c, a putative dioxygenase. We carried out metabolite analysis on a wild-type strain and an Rv3161c mutant strain after exposure to a diaminoquinazoline. The parental compound was found in intracellular extracts from the mutant but not the wild type. A metabolite consistent with a monohydroxylated form was identified in the wild type. These data support the hypothesis that Rv3161c metabolizes diaminoquinazolines in M. tuberculosis.

## Abstract (precis)

The diaminoquinazoline series has good potency against Mycobacterium tuberculosis. Resistant isolates have mutations in Rv3161c, a putative dioxygenase.

## INTRODUCTION

The diaminoquinazoline scaffold has been utilized in the generation of various tool and lead-like compounds for anticancer and antimalarial drug discovery programs (1, 2). A high-throughput screening campaign led to the discovery of a series of diaminoquinazoline (DAQ) compounds that were active against Mycobacterium tuberculosis, with MICs in the submicromolar range (3). We carried out a structure-activity relationship analysis to evaluate the potential of the DAQ series and identified a number of analogs with improved antitubercular activity and good exposure in rat pharmacokinetic studies (4). DAQ compounds had bactericidal activity against replicating and nonreplicating bacteria (4). The target for the DAQ series is not known, but DAQ-resistant isolates have mutations in Rv3161c, a putative dioxygenase (4). Since Rv3161c is not essential and we predict that the mutation would lead to reduced or lower activity, it is unlikely that this is the intracellular target of the series.

Rv3161c is highly induced in M. tuberculosis treated with benzene-containing compounds, such as thioridazine, SRI#967, SRI#9190, and triclosan (5–7). However, triclosan was equally active against wild-type and Rv3161c deletion strains of M. tuberculosis. In addition, strains which overexpressed Rv3161c did not demonstrate triclosan resistance, suggesting that it is not involved in mediating resistance (5).

We hypothesize that DAQ compounds are prodrugs activated by Rv3161c. In order to test this hypothesis, we wanted to determine if DAQ molecules were transformed after uptake by M. tuberculosis. In our previous work, we had only determined the MIC on solid medium using the serial proportion method (8). For compound IDR-0010006, the MIC_99_ for wild-type H37Rv was 6.25 µM, but for the resistant mutants, it was 25 µM (a 4-fold shift) (4). In order to run metabolite identification studies, we determined the MICs for the wild-type and resistant strains of H37Rv in Middlebrook 7H9 liquid medium supplemented with 10% (vol/vol) oleic acid-albumin-dextrose-catalase (OADC) and 0.05% (wt/vol) Tween 80 (7H9-Tw-OADC). Compounds were tested as a 10-point 2-fold serial dilution in 96-well plates, as described previously (8, 9). Growth was measured after 5 days at 37°C and the % growth calculated with respect to the controls. Curves were fitted using the Levenberg-Marquardt algorithm and the IC_90_ calculated as the concentration of compound required to inhibit growth by 90%. We determined the IC_90_s for IDR-0010006 and IDR-0258237 against the wild type and the mutant strain containing the Rv3161c_C115W_ allele (Table 1). The MIC for IDR-0258237 against the wild type was 20 µM, which is consistent with our previous data where IDR-0258237 inhibited the growth of wild-type H37Rv by 98% at 20 µM (4). This was slightly higher than the MIC for IDR-0010006, which was 15 µM. Both compounds were 2.5-fold less active against the mutant strain than against the wild type, which is comparable to the shift seen on solid medium.

**TABLE 1:** Activities of DAQ compounds

H37Rv strain	IC_90_ (μM)^*a*^	
	IDR-0010006	IDR-0258237
Wild type	15 ± 2	22 ± 3
Rv3161c_C115W_ mutant	38 ± 6	59 ± 8

a IC_90_ was determined in liquid medium and is defined as the concentration required to inhibit growth by 90%. The results are the average ± standard deviation from a minimum of 2 experiments.

**FIG 1:**
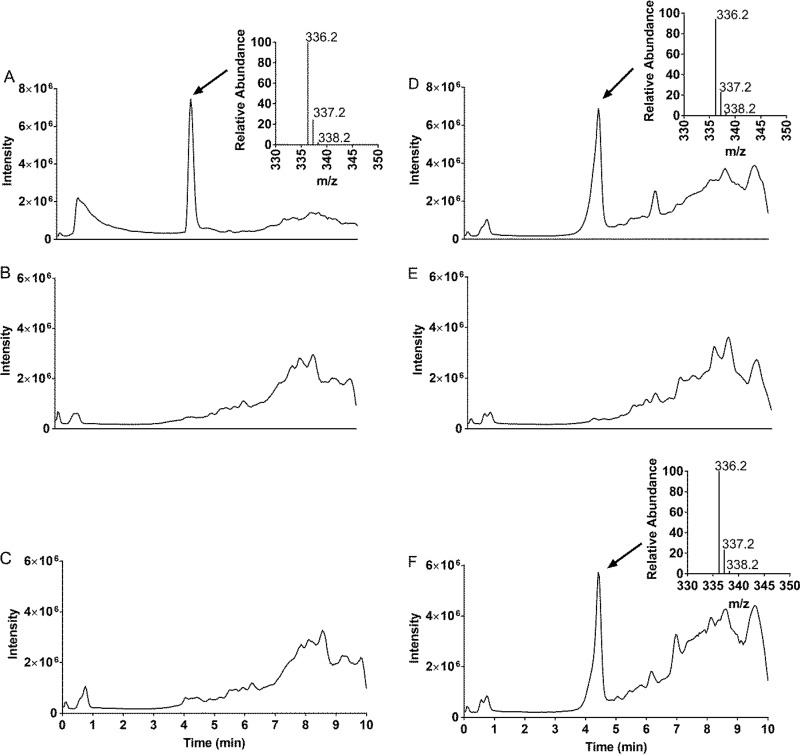
Metabolite analysis. Total ion chromatogram for IDR-0258237 (A) and DMSO only (B). *M. tuberculosis* was treated with 20 µM IDR-0258237 for 24 h (C and D) or for 48 h (E and F). Extracts were subjected to LC-MS. (C and E) Wild-type strain. (D and F) Rv3161c mutant strain. The inset shows the *m/z* peaks associated with the parent ion.

Once we had established the liquid IC_90_, we carried out metabolite analysis using compound IDR-0258237. We selected IDR-0258237 since the MIC was slightly higher for both strains and would enable us to test the compound at a higher concentration; also, we had sufficient compound to treat the large-scale cultures. We first established the settings required to detect the parent compound. Liquid chromatography-mass spectrometry (LC-MS) was carried out on using an Agilent 1100 high-performance LC (HPLC) and G1956B LC/MSD SL mass spectrometer set in positive mode with simultaneous UV-Vis detection at 214 and 254 nm. Buffer A was 0.05% formic acid in water, and buffer B was 0.05% formic acid in acetonitrile. The following solvent gradient was used (time, % buffer B): 0 min, 10%; 30 min, 6.3 min, 95%; 8.1 min, 95%; 8.4 min, 10%; and 10.5 min, 10%. Under these conditions, compound IDR-0258237 eluted at ∼4.5 min, with an *m/z* of 336, which is consistent with the calculated exact mass ([M+H]^+^) of the parent compound (Fig. 1A).

**FIG 2:**
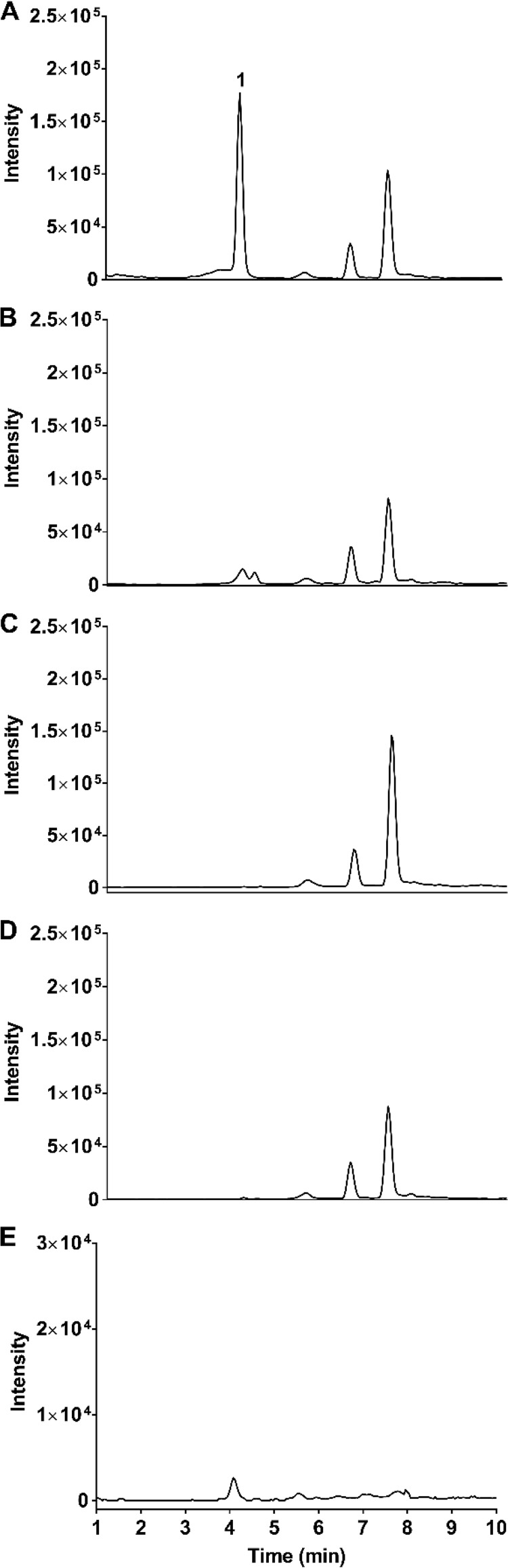
Extracted-ion chromatogram for *m/z* 354. (A) IDR-0258237-treated wild-type strain. (B) IDR-0258237-treated Rv3161c mutant strain. (C) DMSO-treated wild-type strain. (D) DMSO-treated Rv3161c mutant strain. (E) IDR-0258237 only.

Next, we treated wild-type and mutant strains with compound IDR-0258237. M. tuberculosis cultures were grown in 100 ml of 7H9-Tw-OADC in 450-cm^2^ roller bottles at 100 rpm for 5 days at 37°C. Compound IDR-0258237 was added at 20 µM for 24 h or 48h; dimethyl sulfoxide (DMSO) was used as a negative control. Cells were harvested by centrifugation, extracted with an equal volume of 1:1 chloroform-methanol, and incubated at 55°C for 30 min. Samples were reextracted with 1:1 chloroform-methanol and the two extracts pooled. The volume was adjusted to 5 ml with 1:1 chloroform-methanol and refluxed for 16 to 24 h. Pellets were extracted with chloroform, dried, resuspended in 1:1 acetonitrile-water, and subjected to LC-MS analysis (Fig. 1 and 2).

In the wild-type strain, we did not see a peak corresponding to IDR-0258237 in the total ion chromatogram (TIC) (Fig. 1C). In contrast, a peak consistent with IDR-0258237 was observed in extracts from the mutant strain (Fig. 1D). Similar results were observed after 48 h of incubation (Fig. 1E and F).

**FIG 3:**
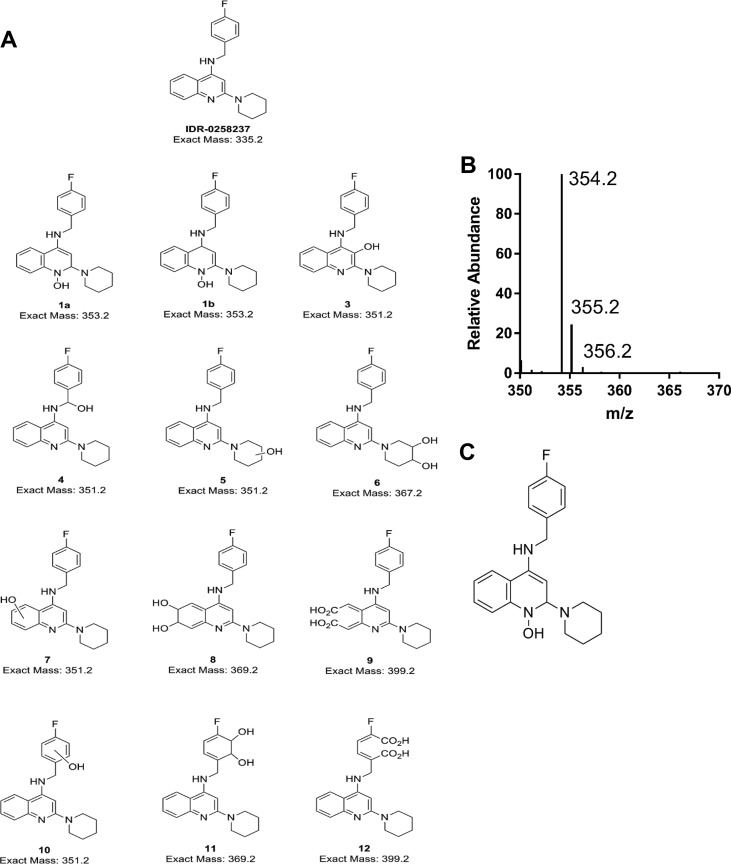
Identification of metabolites. (A) All possible metabolites. (B) MS spectra of the peak 1 detected in the extracted ion chromatogram. (C) Proposed structure of the active metabolite (*m/z* 354.2).

Rv3161c is a putative dioxygenase which might catalyze the incorporation of two oxygen atoms into the substrate. Alternatively, it could function as a monooxygenase and catalyze the introduction of a single oxygen atom. We extracted the ion masses of all potential monohydroxylated and dihydroxylated products from the TIC from all samples (Fig. 2). An ion (peak 1) with a retention time of ∼4.1 min and *m/z* of 354 was observed in the wild-type extracts but not in any of the controls or in the Rv3161c mutant extracts (Fig. 2). The MS properties of this ion are consistent with the addition of a single oxygen atom across the heteroaromatic ring system resulting in the formation of a monohydroxylated DAQ (Fig. 3). Conceivably, dioxygenation followed by the spontaneous elimination of water yielding a monooxygenated product, as described by Carredano et al. (10), could result in the formation of a monohydroxylated DAQ. Alternatively, an epoxidation will also give the observed ion with an exact mass of 354. The formation of an epoxide intermediate has been observed in indoleamine 2,3-dioxygenase-catalyzed reactions (11). We did not detect any other monohydroxylated, dihydroxylated, or cleaved aromatic ring derivatives. LC-tandem mass spectrometry (LC-MS/MS) experiments would be needed for metabolite identification (ID) and confirmation. These results are consistent with Rv3161c catalyzing the modification of DAQ compounds into a metabolite that is active against M. tuberculosis.

A mutation of Rv3161c only led to low-level resistance, although the mutant strain did not appear to metabolize the DAQ compound to any detectable extent. This suggests that both the parent molecule and the metabolite are active but that the metabolite has greater activity against the unknown target. We attempted to synthesize analogs incorporating the predicted hydroxylation, but we were not successful. Therefore, a full characterization of the metabolite(s) and its activity would require large-scale purification directly from M. tuberculosis.

In conclusion, we have determined that a member of the DAQ series is metabolized by wild-type M. tuberculosis but not by a strain containing a mutation in Rv3161c. These data support our hypothesis that the DAQ compounds are biotransformed to more active compounds within the bacterial cell.
